# Update on the Genetic Basis of Sudden Unexpected Death in Epilepsy

**DOI:** 10.3390/ijms20081979

**Published:** 2019-04-23

**Authors:** Monica Coll, Antonio Oliva, Simone Grassi, Ramon Brugada, Oscar Campuzano

**Affiliations:** 1 Cardiovascular Genetics Center, University of Girona-IDIBGI, 17003 Salt, Spain; rbrugada@idibgi.org (R.B.); oscar@brugada.org (O.C.); 2 Section of Legal Medicine, Institute of Public Health, Catholic University, Fondazione Policlinico A. Gemelli, Istituto di Ricovero e Cura a Carattere Scientifico, 00168 Rome, Italy; antonio.Oliva@unicatt.it (A.O.); sgrassifb@gmail.com (S.G.); 3 Centro de Investigación Biomédica en Red de Enfermedades Cardiovasculares (CIBERCV), 28029 Madrid, Spain; 4 Medical Science Department, School of Medicine, University of Girona, 17071 Girona, Spain; 5 Cardiology Service, Hospital Josep Trueta, University of Girona, 17007 Girona, Spain

**Keywords:** SUDEP, epilepsy, genetics

## Abstract

Epilepsy is a common neurological disorder associated with increased morbidity and mortality. Sudden unexpected death in epilepsy, also known as SUDEP, is the main cause of death in patients with epilepsy. SUDEP has an incidence of 1.2 per 1000 person-years in adults and 0.2 per 1000 person-years in children. SUDEP accounts for 8–17% of deaths in patients with epilepsy. It is commonly associated with a history of generalized tonic-clonic seizures, and its risk may be increased by other factors such as postictal electroencephalographic suppression, prone sleeping position, altered heart rate variability, conduction abnormalities, gender, or antiepileptic medications. Recently, electrocardiograms, electroencephalograms, and imaging markers have helped clinicians stratify SUDEP risk and identify patients in need of close monitoring. However, the pathophysiology of SUDEP is likely multifactorial and still unknown. Improving the knowledge of SUDEP incidence, risk factors, and biomarkers can help design and implement effective prevention strategies.

## 1. Introduction

Epilepsy is a common neurological disorder of the central nervous system characterized by recurrent seizures with or without convulsions [1]. Currently, seizures are classified into four groups: simple (status epilepticus), partial (seizures in infants and young adults), complex (generalized tonic-clonic seizures (GTCS)), and unclassified seizures. Those associated with an altered state of consciousness, particularly GTCS and uncontrolled repetitive seizures or status epilepticus, are associated with increased morbidity and mortality, especially in infants and young populations [2,3]. 

In general, patients with epilepsy are two- to three-times more likely to die early than the general population [4]. Several possible causes of death have been reported in patients with epilepsy, including seizure complications, status epilepticus, or even suicide, but the main current cause is sudden unexpected death in epilepsy (SUDEP). Today, SUDEP is defined as a “sudden, unexpected, witnessed or unwitnessed, non-traumatic, and non-drowning death in patients with epilepsy with or without evidence for a seizure, and excluding documented status epilepticus, in which postmortem examination does not reveal a structural or toxicological cause of death” [5].

The concept of SUDEP was first defined in the 1990s based on whether an autopsy was performed and competing factors were present [6,7]. A new classification by Nashef et al. in 2012 helped refine SUDEP definitions and classification [5]. The authors introduced definite SUDEP plus and probable SUDEP plus, which are classifications that consider cases in which the individual had other concomitant conditions. The authors also defined near SUDEP, identifying resuscitated cases after a cardiopulmonary arrest of unidentified origin with survival of >1 h (Table 1). Today, SUDEP is likely underestimated; thus, improved criteria to classify the cause of death would allow more accurate tracking of this condition. Devinsky et al. recently suggested additional clinical and pathological criteria for more consistent and reliable classification of epilepsy-related mortality [8]. Lamberts et al. [9] observed that 62% of SUDEP cases happened between midnight and noon, and that 58% of SUDEP cases were sleep-related. Further, they found that individuals whose deaths were associated with SUDEP were two-times more likely to have nocturnal seizures than those without SUDEP [9]. SUDEP occurs at night likely because of multiple factors, including both situational factors (absence of caregivers) and physiological modifications (circadian rhythms) [10].

## 2. Epidemiology

The current incidence of SUDEP remains undetermined, mainly because SUDEP and epilepsy are frequently underreported as possible or certain causes of death by clinicians and medical examiners [11]. This fact can be (partially) explained by two factors: the lack of accurate and proper knowledge of SUDEP among many practitioners, and the presence of only unspecific—and inconstant—signs in the autopsy (e.g., cortical dysplasia, hippocampal sclerosis, and eosinophilic neuronal change). SUDEP incidence varies depending on the sample population, age range, type of epilepsy, classification systems, diagnosis methods, and recorded cause of death [12,13,14]. Previous estimates indicate that SUDEP risk in young adults (aged 20–45 years) with epilepsy is up to 27-times higher than in the general population of the same age [15].

In 2017, the American Academy of Neurology (AAN) and American Epilepsy Society (AES) published a guideline on SUDEP, reporting an incidence per 1000 epilepsy person-years of 0.22 (95% confidence interval (CI): 0.16–0.31) in children and 1.2 (95% CI: 0.64–2.32) in adults—concluding that patients should be counseled that SUDEP risk is modest in children and small in adults [16]. In 2018, a meta-analysis of 45 cohorts of SUDEP cases estimated that the SUDEP pooled incidence rate was 0.0014 (95% CI: 0.0009–0.0022) events per person-year. In particular, three variables—prospective design, mean patient age, and nature of the study population—explained 22% of the heterogeneity of the incidence rate [17].

As indicated, SUDEP incidence is highest among young adults, while it is considerably lower in the pediatric population. In individuals <14 years of age, SUDEP chiefly accounts for high-risk cases, such as individuals with major neurological impairment or injury [13]. If the onset of epileptic seizures occurs early and never fully remits, the risk of death is about 8% by 70 years of age. However, this risk might vary substantially in different populations, depending on seizure type and extent of seizure control [12]. In 2013, Holst et al. reported that the adjusted hazard ratio for death in young patients with epilepsy was 5.4-times higher than in the general population, and the adjusted hazard ratio for sudden death was 16.3-times higher than in the general population [15]. Consequently, epilepsy is associated with a significantly higher risk of death and sudden death in adults and children. Current AAN guidelines recommend that clinicians inform that SUDEP annually affects 0.2 per 1000 person-years in children with epilepsy and 1.2 per 1000 person-years in adults with epilepsy [16].

## 3. Risk Factors

The main cause of SUDEP remains unknown, and more than one mechanism seems to play a role. Several risk factors specific for SUDEP have been identified in case-control studies on SUDEP and non-SUDEP epilepsy deaths. Although identified risk factors vary due to the heterogeneity of the studies and because models cannot accurately predict the seizure risk for individual patients, some risk factors are recurrent. The strongest and most common risk factor for SUDEP is frequency of seizures, particularly GTCS [13,18,19]. Patients with ≥3 GTCS per month have a 15-fold increased risk of SUDEP. In addition, the presence of GTCS and not being seizure-free for 1–5 years are considered risk factors [16] (Table 2).

Lack of proper/stable antiepileptic drug treatment and polytherapy have also been associated with increased SUDEP risk [12,13,20]. Further, the presence of someone who can provide assistance at night was reported to be protective in one case-control study [21]. Despite the lack of direct evidence, preventive interventions targeting these risk factors could reduce the risk of SUDEP [13].

SUDEP occurs in all ages, although patients who are 20–40 years old have a higher risk of SUDEP [22]. Both earlier age of onset and increased duration of epilepsy are risk factors for SUDEP [23]. SUDEP risk is doubtfully affected by ethnicity and gender, and it seems dependent on the population studied. Despite identified risk factors, however, part of the diagnosed population does not experience SUDEP, suggesting that other mechanisms are involved in the condition [14]. It is important to note that young people’s risk of SUDEP is increased by intellectual disability, structural brain abnormalities, and abnormal neurological assessment [3]. Besides neurological conditions, psychiatric comorbidities also predispose to SUDEP [16].

A recent meta-analysis ranked the 10 leading risk factors derived from key cohort and population-based studies (Table 3) [24].

## 4. Sudden Unexpected Death in Epilepsy (SUDEP) Pathophysiology

Recent studies have extensively investigated the pathophysiology of SUDEP in different groups, proposing four main mechanisms for SUDEP: cardiac dysfunction, respiratory dysfunction, brainstem arousal system dysfunction, and dysregulation in the neurotransmitter and neuromodulator system [28]. Most results suggest a complex and multifactorial model. The MORTality in Epilepsy Monitoring Unit Study (MORTEMUS) study focused on deaths occurring during video electroencephalogram monitoring to ascertain the possible mechanisms of SUDEP on an international scale. The study results suggest that the crucial mechanism leading to SUDEP starts with an early, centrally mediated, severe alteration of both respiratory and cardiac functions after GTCS, which the authors consider as an early postictal neurovegetative breakdown. Depending on the intensity, this mechanism might lead to instant death or cardiorespiratory arrest after several minutes of altered cardiorespiratory function, most likely intensified by profound hypoxia. It has been hypothesized that intrinsic mechanisms leading to or associated with seizure termination are the origin of this centrally mediated neurovegetative breakdown and postictal generalized electroencephalogram (EEG) suppression [23].

## 5. Genetics of SUDEP

Definite evidence has recently emerged concerning genetic susceptibility to SUDEP, suggesting a highly polygenic contribution [18]. A gene associated with SUDEP should include a definite pathogenic alteration that causes epilepsy, increasing SUDEP risk [12]. Numerous neurocardiac genes have been identified as genomic biomarkers of disease severity and outcome, helping predict SUDEP incidence [19]. Moreover, several pathogenic alterations in different genes have been reported to increase SUDEP risk through different pathophysiological mechanisms. These genes encode proteins related to epilepsy and the cardiorespiratory system [18,29]. Until now, few genes have been analyzed in genetic testing (Table 4), but with next-generation sequencing and the low cost of these high-throughput technologies, candidate genes can now be interrogated to explore new genetic causes.

Few studies have examined possible genetic risk factors in SUDEP. In these cases, the limiting factor is sufficient DNA quality of samples collected during the life of the patient or extracted from postmortem blood. However, there is increasing awareness of the importance of collecting blood samples in unexplained sudden death to perform post-mortem genetic testing of main cardiac arrhythmia genes [20]. Further, in 2017, Bagnall et al. performed exome sequencing from degraded DNA derived from fixed postmortem tissues, which identified a de novo *SCN1A* frameshift variant in a patient with SUDEP [20]. This study shows the possibility to analyze historical collections of postmortem fixed tissues from SUDEP cases.

### 5.1. Cardiac Arrhythmia Genes in SUDEP

Epilepsy can have damaging effects on cardiac function, which may play an important role in the pathophysiology of SUDEP [30,31]. Epileptic seizures can induce malignant cardiac arrhythmias, possibly due to seizure-related effects on the autonomic nervous system [20].

Mutations of ion channel genes have a major role in the pathogenesis of several epilepsies, confirming that these are due to the impairment of ion channel function (channelopathies). Voltage-gated channels play an essential role in neuronal excitability and it is not surprising that most mutations associated with epilepsy have been identified in these genes [32].

In 2009, Aurlien et al. identified the first *SCN5A* mutation in a patient with idiopathic epilepsy, suggesting that ion channel mutations are co-expressed in the brain and heart and predispose to both epileptic seizures and cardiac arrhythmias [33]. In 2011, a larger retrospective analysis of autopsies of SUDEP cases in a forensic center in Australia during a 16-year period identified 86 SUDEP cases. The genetic analyses revealed six genetic mutations in voltage-gated ion channel genes *KCNH2* and *SCN5A*, as previously reported in long QT syndrome (LQTS) [34]. Sequencing of a family of four hyperpolarization-activated cyclic nucleotide-gated cation channel genes (*HCN1*, *HCN2*, *HCN3*, and *HCN4*) in the same cohort identified three non-synonymous variants (Phe738Cys and Pro802Ser in *HCN2*, and Gly973Arg in *HCN4*).

The largest genetic study of SUDEP was done by Bagnall et al. in 2016 using exome sequencing-based analysis of 61 SUDEP cases [35]. They analyzed cardiac arrhythmia, respiratory control, and epilepsy genes, identifying four pathogenic and two candidate pathogenic variants of cardiac arrhythmia genes: a de novo *SCN5A* Ile397Val variant, a Gly924Ala and Arg744* nonsense variant in *KCNH2*, and a Tyr662* nonsense variant in *KCNQ1*. The variants in *KCNQ1* and *KCNH2* were previously reported in patients diagnosed with LQTS, and these three variants were absent in more than 60,000 population controls. The de novo variant in *SCN5A* is located in a highly conserved transmembrane domain. All these variants are classified as likely pathogenic for LQTS. In addition, the variants were found in one coronial SUDEP case and three SUDEP cases from the Melbourne Epilepsy Research Centre cohort [20].

In 2016, our group performed next-generation sequencing on a cohort of 14 SUDEP cases using a custom resequencing panel, including genes previously related to SUDEP as well as new candidate genes. Four cases showed rare variants with complete segregation in *SCN1A*, *FBN1*, *HCN1*, *SCN4A*, and *EFHC1*, and one case had a rare variant in *KCNQ1* with incomplete penetrance. In four cases, rare variants were detected in *CACNA1A*, *SCN11A*, *SCN10A*, and *KCNQ1* genes, although no familial segregation was performed due to a lack of DNA from relatives. This study confirmed the link between epilepsy, sudden death, and cardiac disease, and further identified new potential candidate genes for SUDEP: *FBN1*, *HCN1*, *SCN4A*, *EFHC1*, *CACNA1A*, *SCN11A*, and *SCN10A* [36].

The identification of all these gene variants previously associated with LQTS raises some questions about whether the variants are responsible for both epilepsy and sudden death, or if patients with or without gene variants have differing pathophysiology. Further, the identification of LQTS variants in individuals with SUDEP would allow the identification of variants in family members with or without epilepsy who are at risk of arrhythmia. SUDEP is also associated with variants and mutations of neurocardiac channelopathy genes [37]. This evidence could suggest some preventive measures to prevent sudden death, including avoidance of medications that may prolong the QT interval, taking antiarrhythmic drugs such as beta-blockers, and prophylactic interventions for possible lethal cardiac arrhythmias, such as implantable cardioverter defibrillators [20].

### 5.2. Genetic Epilepsy Syndromes and SUDEP Risk

Some genetic epilepsy syndromes have a high risk of SUDEP, and the associated pathogenic variants may be appropriate biomarkers [20]. Pathogenic alterations in *SCN1A* (associated with Dravet syndrome, a genetic epileptic encephalopathy in which a *SCN1A* loss-of-function mutation is found in 80% of cases) or in *SCN8A* (associated with early-infantile encephalopathy) are examples of variants co-expressed in both brain and heart that increase the risk of SUDEP [38]. However, it remains to be determined why these epilepsies caused by genetic variants should have a high risk of SUDEP.

A study by our group that included 20 epilepsy patients with personal or family history of heart rhythm disturbance/cardiac arrhythmia/sudden death identified four missense variants in epilepsy genes *CDKL5*, *CNTNAP2*, *GRIN2A*, and *ADGRV1*, with complete segregation within the family [39]. In 2016, Bagnall et al. [35] performed an exome-based analysis of cardiac arrhythmia, respiratory control, and epilepsy genes in SUDEP. They analyzed 61 SUDEP cases and found one previously described pathogenic mutation and five candidate pathogenic variants in *DEPDC5*. They also identified a variant in sodium channel gene *SCN1A*, related to genetic epilepsy plus febrile seizures and a rare cause of Brugada syndrome, and *SCN2A*, associated with epileptic encephalopathies [35].

### 5.3. Respiratory Genes and SUDEP Risk

The association between neuronal regulation of respiratory function and SUDEP came from functional in vivo models. DBA/1 and DBA/2 (Dilute Brown Non-Agouti) mice are useful animal models to study SUDEP because these mice exhibit generalized convulsive seizures followed by respiratory arrest [40]. Respiratory arrest can be prevented by treatments that activate serotonin (5-HT) receptors, as subtypes of 5-HT help regulate normal respiration [41]. Further, congenital heart hypoventilation syndrome is a disorder characterized by increased frequency of bradyarrhythmia and is mainly caused by expansion of an alanine repeat in *PHOX2B*. This gene could be a good target for SUDEP, although no studied SUDEP cases carry a variant in this gene [42]. Moreover, no genetic variants in respiratory control genes (*ASCL1*, *BDNF*, *EDN3*, *GDNF*, and *RET*) were identified in exome analysis of 61 SUDEP cases [35].

### 5.4. Future Directions

Although considerable progress has been made in SUDEP in recent years, there is much work to be done on the genetic diagnosis of this condition. Several genes responsible for inherited channelopathies are linked to SUDEP, and several recently identified potential candidate genes with positive segregation indicate that a common cardiac neuron gene expression may underlie the basis of sudden death in epilepsy. Although in silico tools are being used to predict the possible pathogenicity and could help classify some variants, it is not recommended that these predictions be used as the sole source of evidence to make a clinical assertion. Moreover, larger cohorts as well as in vivo and in vitro studies will be required to ascertain the pathogenicity of these variants. Molecular genetic screening also needs to become an inherent part of the post-mortem examination in cases labeled as SUDEP. This genetic testing will enhance the ability to screen SUDEP victims’ family members, who may be at-risk carriers of fatal cardiac disorders. Genetic interpretation requires multidisciplinary groups, including geneticists working together with clinicians, forensic pathologists, and genetic counselors. To best manage families, close interdisciplinary collaboration is essential.

## 6. New Biomarkers

Although all individuals with epilepsy are at risk of SUDEP, the risk is not uniform across patient groups. Biomarkers are needed to identify the individual risk of SUDEP and thus personalize preventive interventions. Biomarkers might address innate risk factors of SUDEP, such as the genetic profile, as well as acquired risk factors (some of which might change over time), such as the level of post-GTCS brainstem dysfunction [43]. Three main tools are currently used: electrocardiogram (ECG), electroencephalogram (EEG), and imaging biomarkers (MRI and fMRI).

In 2013, Scorza et al. [44] first mentioned the term microRNA in SUDEP; microRNAs (miRNAs) are noncoding RNA molecules that play a critical role in gene expression regulation. These molecules are involved in important biological processes, are potential biomarkers for cardiovascular disorders, and can be detected in serum or plasma in a remarkably stable form. The authors suggested that expression pattern modifications of circulating miRNAs may be related to abnormal underlying cardiovascular processes, and could be identified and used as biomarkers for SUDEP [44]. In 2017, De Matteis et al. [45] presented a case of SUDEP in comparison with 10 autopsies of traumatic or asphyxia deaths. The authors analyzed expression profiles of several miRNAs (miR-301a-3p, miR-194-5p, miR-30b-5p, mIR-342-5p, and miR-4446-3p) from the plasma and temporal lobe, and identified a significant up-regulation of miR-301a-3p in the plasma (2.3-fold) and hippocampus (3.2-fold) of the SUDEP case compared with controls [45]. However, this potential biomarker still needs to be confirmed by additional cases.

## 7. Prevention of SUDEP

The main purpose of unravelling the incidence, risk factors, and biomarkers of SUDEP is to design and implement effective prevention strategies. Education is the first measure of prevention—treating physicians, primary care physicians, and neurologists must inform patients about risk factors for morbidity and mortality associated with medically refractory epilepsy, and about the importance of adhering to therapy and avoiding risky and/or unhealthy behaviors (e.g., sleeping in prone position, insufficient sleep, excessive drinking) [30]. Reported preventive measures include effective seizure control, nocturnal supervision, seizure monitoring, devices to protect the airway (e.g., anti-suffocation pillows), selective serotonin reuptake inhibitors, and other medications (caffeine and other adenosine antagonists have been proposed as hypothetical treatments) [28].

However, the evidence supporting these interventions is not robust [46,47]. The most effective prevention of SUDEP is likely complete remission of GTCS [48]. Usually, SUDEP occurs during sleep without supervision. Therefore, a preventive measure could involve using seizure and apnea detection devices to recognize the initial step of SUDEP and thus start resuscitation procedures as soon as possible. Cardiorespiratory resuscitation strategies have also been proposed as preventive measures for SUDEP. In 2008, Strzelczyk et al. described that implantation of pacemakers and defibrillators prevented ictal asystole in temporal lobe epilepsy patients [49].

## 8. Conclusions

SUDEP is the main cause of epilepsy-related premature mortality, especially in younger populations. Different types of epilepsy populations complicate the identification of SUDEP cases and, consequently, of its current incidence. Therefore, systematic methods to improve identification and better understand the causes of this condition are needed. Advancing our knowledge of SUDEP pathophysiologic mechanisms is the first step to reduce its incidence and elaborate new preventive strategies. At the same time, diffusion of knowledge about SUDEP must be supported and amplified inside scientific and medical communities to improve the quantity and quality of data available for research. In addition, biomarkers such as ECG, EEG, and imaging markers will help clinicians further stratify SUDEP risk and identify patients who need close monitoring. The identification of additional risk factors and biomarkers will help develop new preventive interventions for patients and their families. Ultimately, education of both families and clinicians as well as basic and clinical research and development of new treatments are needed to reduce the risk of SUDEP.

## Figures and Tables

**Table 1:** Proposed sudden unexpected death in epilepsy (SUDEP) definition and classification [5].

Classification	Definition
Definite sudden unexpected death in epilepsy (SUDEP)	Sudden, unexpected, witnessed or unwitnessed, non-traumatic, and non-drowning death that occurs in benign circumstances in an individual with epilepsy, with or without evidence for a seizure, and excludes documented status epilepticus, in which post-mortem examination does not reveal a cause of death.
Definite SUDEP plus	Death satisfying criteria for definite SUDEP, if a concomitant condition other than epilepsy is identified before or after death, if the death might have been due to the combined effect of both conditions, and if autopsy or direct observations or recording of the terminal event did not prove the concomitant condition to be the cause of death.
Probable SUDEP or probable SUDEP plus	Same definition as definite SUDEP or SUDEP plus, but without autopsy.
Possible SUDEP	A competing cause of death is present.
Near-SUDEP or near-SUDEP plus	A patient with epilepsy who survives resuscitation for more than an hour after cardiorespiratory arrest and has no structural cause identified after investigation.
Not SUDEP	A clear alternative cause of death is identified.
Unclassified	Incomplete information available; impossible to classify.

**Table 2:** SUDEP risk factors.

Risk	Factor
High	Frequency of generalized tonic-clonic seizures (GTCS)
Moderate	Presence of GTCS
	Not being seizure-free for 1–5 years
	Not adding an antiepileptic drug when patients are medically refractory
	Nocturnal supervision (risk reduction)
	Use of nocturnal listening device (risk reduction)

**Table 3:** Principal risk factors for SUDEP (adapted from Reference [24]).

Risk Factor	Weighted Log Odds Ratio (Adjusted Log OR ÷ Standard Error (SE))	Reference
>3 GTCS per year (versus 0 seizures)	12.10	Hesdorffer et al. [25]
>13 Seizures of any type in the past year (versus 0–2 seizures)	4.20	Nilsson et al. [26]
No AED * treatment (versus 1–2 AEDs)	3.78	Langan et al. [21]
3 AEDs (versus 1)	3.24	Nilsson et al. [26]
≥3 GTCS in past year (versus 0)	3.04	Walczak et al. [27]
11–20 GTCS in the past 3 months (versus 0–5)	2.39	Langan et al. [21]
Age at onset of 0–15 years (versus >45 years)	2.29	Nilsson et al. [26]
IQ <70	2.23	Walczak et al. [27]
3–5 changes in AEDs per year (versus 0)	2.16	Nilsson et al. [26]
>3 AEDs in the last visit (versus 0–2)	1.96	Walczak et al. [27]

* AED: antiepileptic drugs.

**Table 4:** Genes associated with SUDEP (adapted from Reference [20]).

Gene	Description	Evidence for Association with SUDEP
*SCN1A*	Sodium Voltage-Gated Channel Alpha Subunit 1	Animal model; de novo variants found in SUDEP cases
*SCN2A*	Sodium Voltage-Gated Channel Alpha Subunit 2	De novo variants found in SUDEP cases
*SCN5A*	Voltage-Gated Sodium Channel Subunit Alpha Nav1.5	De novo variant found in SUDEP case
*SCN8A*	Sodium Voltage-Gated Channel Alpha Subunit 8	Animal model; de novo variants found in SUDEP cases
*KCNA1*	Potassium Voltage-Gated Channel Subfamily A Member 1	Animal model; variant found in SUDEP case
*KCNQ1*	Voltage-Gated Potassium Channel Subunit Kv7.1	Variants found in SUDEP cases
*KCNH2*	Voltage-Gated Potassium Channel Subunit Kv11.1	Variants found in SUDEP cases
*DEPDC5*	DEP Domain-Containing Protein 5	De novo variants found in SUDEP cases

## Abbreviations

SUDEP	Sudden unexpected death in epilepsy
GTCS	Generalized tonic-clonic seizures
AED	Antiepileptic drugs
LQTS	Long QT syndrome
ECG	Electrocardiogram
EEG	Electroencephalogram
